# Achievement and Persistence of MRD Negativity with Anti-CD38-Based Regimens in Newly Diagnosed Multiple Myeloma: A Systematic Review and Network Meta-Analysis

**DOI:** 10.3390/cancers18213573

**Published:** 2026-11-06

**Authors:** Giulio Caridà, Luca Scalise, Antonio Mosca, Laura De Fazio, Martina Costa, Matteo Molica, Marco Rossi

**Affiliations:** 1 Haematology Unit, Renato Dulbecco Hospital, 88100 Catanzaro, Italy; g.carida@unicz.it (G.C.); martinamcosta@gmail.com (M.C.); 2 Department of Hematology-Oncology, Azienda Ospedaliera Renato Dulbecco, 88100 Catanzaro, Italy

**Keywords:** network meta-analysis, measurable residual disease, MRD, sustained MRD

## Abstract

**Background/Objectives:** Anti-CD38 monoclonal antibody-based quadruplets have increased measurable residual disease (MRD) negativity rates in newly diagnosed multiple myeloma, questioning the incremental benefit of upfront autologous stem cell transplantation (ASCT). **Methods:** We performed a systematic review and frequentist random-effects network meta-analysis (NMA) of randomized trials reporting MRD negativity by treatment arm; sustained MRD (sMRD) negativity was analyzed by pairwise meta-analysis. **Results:** Seventeen trials (6293 patients; 13 strategies) were included. Anti-CD38–KRd (SUCRA 89.1%), anti-CD38–VRd + ASCT (88.0%) and anti-CD38–KRd + ASCT (86.5%) ranked highest for MRD negativity, with no statistically significant difference among them. No comparison between a strategy with and without ASCT reached statistical significance (VRd + ASCT vs. VRd: OR 1.72, 95% CI 0.95–3.10). There was no evidence of inconsistency, and rankings were consistent across sensitivity analyses. Adding an anti-CD38 antibody to the same backbone increased the odds of sMRD negativity in both transplant-eligible (OR 3.05, 95% CI 1.32–7.03) and transplant-ineligible populations (3.26, 2.18–4.88; *p* for interaction = 0.88). No sMRD data allowed a comparison between transplant and non-transplant quadruplet strategies. **Conclusions:** Anti-CD38-based quadruplets offer the highest probability of achieving MRD negativity and improve its persistence regardless of transplant eligibility, whereas the incremental contribution of ASCT in the quadruplet era remains uncertain.

## Simple Summary

Four-drug combinations including an anti-CD38 antibody (daratumumab or isatuximab) can improve measurable residual disease (MRD) negativity rates in multiple myeloma newly diagnosed patients. To date, the additional benefit of stem cell transplantation to these regimens is debated. We combined data from 17 randomized trials including 6293 patients. Four-drug regimens combining an anti-CD38 antibody with carfilzomib-lenalidomide-dexamethasone (with or without transplantation), or with bortezomib-lenalidomide-dexamethasone followed by transplantation, were the most likely to achieve MRD negativity, with no clear difference among them. Adding an anti-CD38 antibody also roughly tripled the chance of remaining MRD-negative for at least one year, both in patients eligible and in those ineligible for transplantation. Current data do not establish whether transplantation further improves these deep responses when four-drug regimens are used; dedicated trials are needed to answer this question.

## 1. Introduction

Multiple myeloma (MM) is a hematological disease characterized by the clonal proliferation of malignant plasma cells, accounting for approximately 1% of all malignancies and 15–20% of hematological cancers, with an estimated 106,000–121,000 deaths worldwide each year [1]. Although MM remains substantially incurable, its prognosis has improved over the past two decades as a result of the introduction of novel drugs and their incorporation into effective combination regimens [2]. Indeed, MM treatment switched from chemo-based to immune-based strategies, relying on immunomodulatory drugs (IMiDs), such as thalidomide and lenalidomide, proteasome inhibitors (PIs), bortezomib and carfilzomib, and anti-CD38 monoclonal antibodies, including daratumumab and isatuximab.

CD38 shows a high and uniform expression in malignant plasma cells, making it a good therapeutic target. Daratumumab, a human IgG1κ antibody, and isatuximab, a chimeric IgG1 antibody, target different binding sites and also work in a complementary way through antibody-mediated cytotoxicity and phagocytosis as well as by draining immune-suppressive CD38-positive cells through immunomodulation; isatuximab also sometimes causes apoptosis. Both drugs were first approved for relapsed cases. The two drugs are now being utilized in combination with other drugs in the treatment of both transplantable and non-transplantable patients.

On these bases, the updated treatment landscape is composed of highly active triplet and quadruplet drug regimens capable of inducing unprecedented rates of deep responses [3]. These advances have occurred in both autologous stem cell transplant (ASCT)-eligible (TE) and -ineligible (TI) populations, thus reducing the traditional therapeutic divide between these two clinical settings.

Nevertheless, international guidelines [4] still indicate high-dose melphalan followed by ASCT as a standard component of frontline treatment for fit, TE patients, owing to its capacity to achieve substantial cytoreduction and prolong disease control. However, the unprecedented efficacy of contemporary pharmacological combinations [5] has fueled the debate regarding the magnitude of the additional benefit provided by upfront ASCT and whether this procedure should be routinely performed as early consolidation or selectively deferred. Accordingly, several recent randomized clinical trials (RCTs) have investigated the efficacy and safety of modern combinations in both TE and TI newly diagnosed MM (NDMM) patients [6], providing an increasingly complex body of evidence across treatment strategies that differ in drug backbone, treatment intensity and use of ASCT. Measurable Residual Disease (MRD) negativity is the most suitable common endpoint for comparing the depth of response produced by different strategies [7,8]. MRD negativity is consistently associated with longer PFS and OS across all patient subgroups, being a good intermediate endpoint to predict earlier efficacy signal than PFS or OS, while sustained MRD negativity (sMRD) may be more informative than a single negative assessment [9].

Although the increasing efficacy of antibody-based combinations has raised uncertainty regarding the incremental benefit of upfront ASCT, randomized evidence comparing ASCT with quadruplet-based non-transplant strategies is limited to a single trial in which randomization was conditional on post-induction MRD status, and no trial has compared these strategies for sustained MRD negativity.

Given the absence of comprehensive head-to-head evidence, network meta-analysis (NMA) provides a suitable framework for integrating direct and indirect comparisons across available RCTs. We therefore performed a systematic review and network meta-analysis to compare and rank frontline treatment strategies for newly diagnosed MM according to their probability of achieving MRD and sMRD negativity.

## 2. Methods

This systematic review was performed in accordance with the PRISMA (Preferred Reporting Items for Systematic Reviews and Meta-Analyses) 2020 guidelines [10] and has not been registered. The completed PRISMA-NMA checklist is provided in Supplementary S1.

A comprehensive literature search was performed in PubMed/MEDLINE, Embase, and the Cochrane Central Register of Controlled Trials. The proceedings of major international hematology and oncology meetings, including the American Society of Hematology, the European Hematology Association, and the American Society of Clinical Oncology, were also searched for relevant unpublished or recently presented studies. The literature search was performed in October 2026 and covered records published up to 31 July 2026. For trials available only as conference abstracts (ADVANCE, COBRA, AGMT-MM04), data were extracted from the most recent presentation; all other trials were extracted from full peer-reviewed publications. The search strategy combined controlled vocabulary and free-text terms related to “multiple myeloma,” “newly diagnosed multiple myeloma,” “measurable residual disease,” “minimal residual disease,” “autologous stem cell transplantation,” “daratumumab,” “isatuximab,” “bortezomib,” “carfilzomib,” “lenalidomide,” and “randomized controlled trial.” The complete search strategy for each database is provided in the Supplementary S2.

Two investigators independently screened titles and abstracts, assessed potentially eligible full-text articles, and selected the studies for inclusion. Disagreements were resolved by discussion and consensus. When multiple publications or conference presentations reported results from the same trial, the most complete and updated dataset was used, while earlier reports were consulted to retrieve additional methodological information. Data were extracted by two investigators using a standardized form, and discrepancies were resolved by consensus.

Studies were eligible if they met the following criteria: (1) randomized controlled phase II or III trials; (2) inclusion of adult patients with newly diagnosed MM; (3) evaluation of a systemic frontline treatment strategy; (4) comparison of at least two treatment strategies that could be incorporated into the evidence network; and (5) reporting of MRD negativity for each randomized treatment group. Trials enrolling either TE or TI patients were included. Eligible interventions comprised immunomodulatory drug–, proteasome inhibitor–, and anti-CD38 monoclonal antibody-based regimens, administered with or without high-dose melphalan followed by autologous stem cell transplantation (ASCT).

The exclusion criteria were: non-randomized or single-arm studies; trials conducted exclusively in patients with relapsed or refractory MM; studies evaluating subsequent-line treatment; trials without extractable MRD-negativity data for the randomized groups; retrospective comparisons and studies with insufficiently reported methodology. Regimens including monoclonal antibodies other than anti-CD38 agents (e.g., elotuzumab), immune checkpoint inhibitors, bispecific antibodies or CAR-T cells were not considered eligible interventions.

Studies were excluded from the quantitative synthesis if their treatment arms could not be connected to the principal evidence network or if MRD outcomes were not sufficiently reported, extractable, or compatible with the prespecified outcome definition.

Daratumumab- and isatuximab-containing regimens were grouped into a common anti-CD38 node in the primary analysis; modeling daratumumab and isatuximab as separate nodes was explored as a sensitivity analysis including all trials.

The primary outcome was MRD negativity, defined according to the criteria, analytical method, and sensitivity threshold adopted by each trial. MRD was generally assessed by next-generation flow cytometry or next-generation sequencing at thresholds of at least 10^−5^ and 10^−6^.

The timing, assay methodology, sensitivity threshold, and denominator used to calculate MRD-negativity rates were recorded for each trial.

Sustained MRD (sMRD) negativity was defined as MRD negativity maintained for at least 12 months, as reported by each trial, at a 10^−5^ threshold in the intention-to-treat population. As no trial reported sMRD for the comparisons linking transplant-based, carfilzomib-based and non-transplant strategies, the sMRD evidence formed disconnected components and a network meta-analysis was not feasible. The effect of adding an anti-CD38 antibody to the same backbone regimen was therefore evaluated by pairwise meta-analysis of trials comparing an anti-CD38-containing regimen with the corresponding regimen without anti-CD38. For MRD negativity NMA, MRD was analyzed as a binary outcome.

For each randomized treatment group, the number of patients achieving MRD negativity and the total number of patients included in the relevant analysis population were extracted. Comparative treatment effects were expressed as odds ratios (ORs) with corresponding 95% confidence intervals (CIs). An OR greater than 1 indicated a higher probability of achieving MRD negativity for the treatment reported in the numerator.

For sMRD pairwise meta-analysis, ORs were pooled using a random-effects model (DerSimonian–Laird), with TE and TI/transplant-deferred populations analyzed as subgroups and compared by a test for subgroup differences. Heterogeneity was quantified with the I^2^ statistic.

The risk of bias of the included randomized trials was assessed using the Cochrane Risk of Bias tool [11]. The confidence in the results of the network meta-analysis was assessed with the CINeMA framework (Confidence in Network Meta-Analysis; cinema.ispm.unibe.ch) [12,13], which evaluates within-study bias, reporting bias, indirectness, imprecision, heterogeneity and incoherence.

The analysis was conducted using Stata, version 17 (StataCorp, College Station, TX, USA), with the network suite and the mvmeta and metan packages. The Stata code used for all analyses is provided in Supplementary S3. Confidence in the evidence was assessed with the CINeMA web application (Institute of Social and Preventive Medicine, University of Bern, Bern, Switzerland).

Relative treatment rankings were estimated using the surface under the cumulative ranking curve (SUCRA) and the probability of each strategy being the best-performing treatment. SUCRA values range from 0% to 100%, with higher values indicating a greater probability that a treatment ranks among the most effective strategies for achieving MRD negativity. Ranking results were interpreted together with the magnitude and precision of the relative treatment effects and were not considered evidence of statistically significant superiority in isolation [14]. Network meta-analysis was performed within a frequentist framework using a random-effects model, with restricted maximum likelihood (REML) estimation and a common between-study heterogeneity (τ) assumed across comparisons. Log odds ratios were derived from arm-level numbers of MRD-negative patients and analyzed patients. A continuity correction of 0.5 was added to all cells of trials with a zero-event arm (EMN20). The three-arm FORTE and GEM2017FIT trials were modeled as multi-arm studies, accounting for the correlation between their comparisons. Treatment rankings were estimated from 5000 simulations of the network estimates (fixed random seed).

Sensitivity analyses were performed using a fixed-effect model, excluding GMMG-HD7 (MRD assessed after induction, before ASCT), restricting to trials using a 10^−5^ MRD threshold, excluding abstract-only data, analyzing transplant-eligible/deferred and transplant-ineligible trials as separate networks, modeling daratumumab and isatuximab as separate nodes, and performing leave-one-trial-out analyses where exclusion did not disconnect the network.

Global inconsistency was assessed using the design-by-treatment interaction model, and local inconsistency by node-splitting, comparing direct and indirect estimates for each comparison informed by both sources of evidence. Publication bias could not be formally assessed with comparison-adjusted funnel plots, as no comparison was informed by more than three trials.

The results of all network comparisons were summarized in league tables reporting ORs and corresponding 95% CIs. A difference was considered statistically significant when the 95% CI did not include 1.

## 3. Results

### 3.1. Study Selection and Characteristics

After removal of duplicates, 4119 records were screened, and 17 randomized controlled trials were ultimately included in the systematic review and quantitative synthesis (Figure 1), comprising a total of 6293 patients. The queries of the systematic review are reported in Supplementary S2. The included trials were ADVANCE [15], AGMT-MM04 [16], BENEFIT [17,18], CARDAMON [19], CEPHEUS [20], COBRA [21], DETERMINATION [22], EMN20 [23], FORTE [24,25], GEM2017FIT [26], GMMG-HD7 [27], GRIFFIN [28], IFM 2009 [29,30], IMROZ [31], IsKia [32], MAIA [33], and PERSEUS [5]. The characteristics of each trial are summarized in Table 1; all treatment strategies are listed in Table 2. The contribution of each trial of each node is reported in Supplementary S4.

The evidence network comprised 13 treatment nodes (Figure 2): eight strategies without ASCT (anti-CD38–KRd, anti-CD38–VRd, anti-CD38–Rd, KRd, KCd, VRd, VMP → Rd and Rd) and five incorporating ASCT (anti-CD38–KRd + ASCT, anti-CD38–VRd + ASCT, KRd + ASCT, KCd + ASCT and VRd + ASCT). DETERMINATION, IFM 2009, FORTE and CARDAMON directly compared transplant and non-transplant approaches. The inclusion of EMN20 (KRd vs. Rd) created a closed loop (Rd–KRd–VRd–anti-CD38–VRd–anti-CD38–Rd) informed by independent trials. As GMMG-HD7 enrolled TE patients who subsequently underwent ASCT, its treatment arms were assigned to the VRd + ASCT and anti-CD38–VRd + ASCT nodes, although MRD was assessed after induction and before ASCT; the impact of this choice was explored in a sensitivity analysis (Supplementary S5).

### 3.2. Risk of Bias

Risk of bias was low in all domains for 10 of the 17 trials (BENEFIT, CARDAMON, CEPHEUS, FORTE, GMMG-HD7, GRIFFIN, IMROZ, IsKia, MAIA and PERSEUS). Five trials had at least one domain at unclear risk, mainly because data were available only as conference abstracts or interim analyses (ADVANCE, COBRA), MRD was assessed in a subset of patients (IFM 2009), allocation concealment was not reported (EMN20, GEM2017FIT) or the trial was terminated early (EMN20). Two trials were at high risk of attrition bias: DETERMINATION, in which MRD was assessed in a subset of patients, and AGMT-MM04, which reported an interim per-protocol analysis. The risk-of-bias assessment is summarized in Supplementary S6.

### 3.3. Network Geometry

Several randomized trials reporting MRD outcomes were not included in the quantitative synthesis because their treatment arms could not be connected to the evidence network through a common comparator: CASSIOPEIA (D-VTd vs. VTd, both followed by ASCT), ALCYONE (D-VMP vs. VMP) and EMN02/HO95 (VMP vs. high-dose melphalan and ASCT after VCd induction, with MRD assessed by flow cytometry in a subset of patients only). SWOG S0777 (VRd vs. Rd) and ENDURANCE (KRd vs. VRd) were potentially connectable but did not report MRD negativity by randomized arm. In MIDAS (IFM 2020-02), randomization to ASCT or Isa-KRd consolidation occurred after Isa-KRd induction and was stratified by post-induction MRD status; as both the randomized population and the outcome were conditional on prior MRD results, this trial was not considered transitive with the other studies and is addressed in the Discussion.

There was no evidence of global inconsistency (design-by-treatment interaction: χ^2^ = 3.71, df = 2, *p* = 0.16). Node-splitting did not identify significant disagreement between direct and indirect evidence for any comparison (all *p* ≥ 0.17). The largest discrepancy involved the KRd vs. Rd comparison, for which the direct estimate from EMN20 was large and imprecise because of zero events in the Rd arm.

### 3.4. MRD Negativity: Network Meta-Analysis

Treatment-ranking analyses indicated that anti-CD38-containing quadruplet strategies were most likely to achieve MRD negativity. Anti-CD38–KRd achieved the highest SUCRA value (89.1%) but not the highest probability of being the best-performing treatment (27.6%). Anti-CD38–VRd plus ASCT ranked second, with a SUCRA value of 88.0% and a 36.9% probability of ranking first, followed by anti-CD38–KRd plus ASCT, with a SUCRA value of 86.5% and a 34.1% probability of being the best treatment.

Among the remaining strategies, KRd plus ASCT ranked fourth (SUCRA 70.8%), followed by KRd (65.3%) and Anti-CD38–VRd (57.4%).

VRd plus ASCT and KCd plus ASCT occupied lower positions, with SUCRA values of 51.9% and 45.6%, respectively. VRd without ASCT ranked next, with a SUCRA value of 29.1%. The lowest-ranked strategies were anti-CD38–Rd (SUCRA 15.8%) and Rd (SUCRA 0.3%). SUCRA values are reported in Table 3.

The addition of an anti-CD38 antibody was associated with higher odds of MRD negativity when added to VRd + ASCT (OR 2.84, 95% CI 1.80–4.48), KRd (1.97, 1.10–3.52) and VRd (1.95, 1.13–3.38). No comparison between a strategy with ASCT and the corresponding strategy without ASCT was statistically significant: VRd + ASCT vs. VRd, OR 1.72 (0.95–3.10); KRd + ASCT vs. KRd, 1.18 (0.52–2.66); KCd + ASCT vs. KCd, 2.10 (0.87–5.05); anti-CD38–VRd + ASCT vs. anti-CD38–VRd, 2.50 (0.99–6.32).

Overall, the three highest-ranked strategies accounted for almost the entire probability of being the best-performing treatment (98.6%); however, none of the pairwise comparisons among them reached statistical significance (Figure 3).

### 3.5. Inconsistency and Sensitivity Analyses

Results were robust across sensitivity analyses (Supplementary S5). The three anti-CD38-based quadruplets occupied the top three positions in the fixed-effect model, after exclusion of GMMG-HD7, when restricting to the 10^−5^ threshold, in the TE/ASCT-deferred network, and in all leave-one-out analyses in which the network structure was preserved. Anti-CD38–VRd + ASCT ranked first when GMMG-HD7 or ADVANCE was excluded, and exclusion of GMMG-HD7 reduced τ from 0.35 to 0.23. With separate antibody nodes, daratumumab–VRd + ASCT, daratumumab–KRd and isatuximab–KRd + ASCT ranked highest. When COBRA was excluded (abstract-only and leave-one-out analyses) and in the TI network, carfilzomib-based strategies were connected only through EMN20, which had zero events in the Rd arm, yielding extremely imprecise estimates that were not interpreted. The OR for VRd + ASCT vs. VRd was stable across analyses (1.65–1.81), but its 95% CI excluded 1 only in the fixed-effect model and in some sensitivity analyses with lower estimated heterogeneity.

### 3.6. Sustained MRD Negativity

As sMRD data could not be connected into a single network, sMRD was analyzed by pairwise meta-analysis of the six trials comparing an anti-CD38-containing regimen with the same backbone without anti-CD38 (PERSEUS, GRIFFIN, IsKia, IMROZ, CEPHEUS and MAIA; Figure 4). The addition of an anti-CD38 antibody was associated with higher odds of sMRD negativity (≥12 months, 10^−5^) in both TE (OR 3.05, 95% CI 1.32–7.03) and TI/transplant-deferred populations (OR 3.26, 95% CI 2.18–4.88), with no evidence of a difference between settings (*p* for interaction = 0.88; overall OR 3.18, 95% CI 2.12–4.76). Heterogeneity was substantial (I^2^ = 79%) and mainly driven by IsKia, in which the control arm (KRd plus ASCT) already achieved high sMRD rates (OR 1.33, 95% CI 0.83–2.12). The only randomized comparison of sMRD with versus without ASCT was provided by FORTE, in which 1-year sustained MRD negativity was 47% with KRd plus ASCT versus 35% with KRd12 (OR 1.68, 95% CI 1.07–2.65).

### 3.7. Certainty of Evidence

Confidence in the evidence was high for anti-CD38–Rd versus Rd and moderate for anti-CD38–VRd + ASCT versus VRd + ASCT. It was low for anti-CD38–VRd versus VRd (because of heterogeneity) and for anti-CD38–KRd and anti-CD38–VRd + ASCT versus VRd (because of within-study bias and incoherence). Confidence was very low for all comparisons between strategies with and without ASCT and among the three top-ranked strategies, mainly because of imprecision (Supplementary S7).

## 4. Discussion

The main finding of the present NMA is the highest ranking of anti-CD38-containing quadruplet strategies for MRD negativity. Anti-CD38–KRd ranked first and showed a ranking profile broadly comparable to anti-CD38–VRd + ASCT treatment regimen. Although an NMA for sMRD negativity was not possible due to the lack of data, a pairwise meta-analysis showed that the addition of an anti-CD38 antibody was associated with approximately threefold higher odds of sMRD negativity, consistently in TE and TI populations.

The role of adding ASCT was evaluated in the phase 3 MIDAS (IFM 2020-02) trial [34], in which Isa-KRd plus ASCT was compared to Isa-KRd alone. Among the 485 patients who were MRD-negative (10^−5^) after induction, those randomized to ASCT followed by two cycles of Isa-KRd and those receiving six further cycles of Isa-KRd achieved similar MRD negativity rates at 10^−6^ after consolidation (86% vs. 84%; *p* = 0.64). In MRD-positive patients, tandem ASCT did not improve MRD negativity compared with single ASCT (32% vs. 40%; *p* = 0.31). Neither MIDAS nor our analysis can yet establish whether ASCT improves the persistence of MRD negativity or long-term outcomes: progression-free survival data from MIDAS are immature, and no trial reported sMRD for a transplant versus non-transplant quadruplet comparison.

MRD negativity is an intermediate endpoint, and PFS and OS were not analyzed in this NMA. In DETERMINATION and IFM 2009, upfront ASCT after VRd induction prolonged PFS without improving OS. In the original trials, the addition of an anti-CD38 antibody improved PFS in PERSEUS, GRIFFIN, IMROZ, CEPHEUS and MAIA, and OS in MAIA, whereas survival data for anti-CD38–KRd (ADVANCE, GEM2017FIT) and anti-CD38–KRd + ASCT (IsKia) remain immature. Whether the small, non-significant differences in MRD negativity between transplant and non-transplant strategies observed here translate into differences in long-term outcomes cannot be inferred from our data.

These findings provide additional insights into the ongoing debate on whether upfront ASCT can efficaciously improve the outcome of TE-NDMM patients in the era of highly active multidrug therapy. Furthermore, these data highlight the distinction between achieving MRD negativity and MRD negativity persistence over time, but the real effect size of ASCT within contemporary frontline treatment strategies for NDMM must be further investigated. Furthermore, MRD has to be intended as a dynamic outcome: a single MRD-negative assessment captures the maximum depth of response documented at a specific time point, providing the essential information on the sensitivity of malignant plasma cells to the treatment. sMRD negativity incorporates the additional parameter of temporal dimension and may therefore more reliably identify prolonged suppression and/or clearance of the residual malignant clone. The debate on the role of MRD negativity as surrogate marker of outcome has been unveiled by Munshi et al., who performed a large MA based on 93 publications [35]. In this work, data on sMRD were not widely available to perform MA, although the authors remark its likely relevance in future treatment decision making. The different weight between MRD and sMRD emerged more clearly when lenalidomide became part of first-line maintenance in NDMM patients. Indeed, the Myeloma XI trial [36] showed that lenalidomide increased conversion to MRD negativity and extended MRD negativity time lapse compared to no maintenance, improving the PFS rate likelihood. The dynamic nature of the MRD tool has been further proven in retrospective clinical trial and real-life studies [37,38]. Further proof of the potential role of the sMRD tool in clinical decision making is the prospective study from Terpos et al. [39], where lenalidomide maintenance could be interrupted if sMRD was preserved over 3 years of treatment. These data raise the question of which could be the factors that influence the difference between MRD achievement and persistence over time.

The ranking observed in our analyses indicates that some regimens are particularly effective in inducing MRD negativity, whereas others may be more effective in maintaining it through consolidation, ASCT, or prolonged maintenance. sMRD rates also depend on the type and duration of maintenance and on the timing of MRD assessments, which differed across trials. Overall, these data support the concept that MRD and sMRD negativity should be considered as distinct endpoints and indicate that the incorporation of standardized MRD assessment over defined time points as the main surrogate endpoint for outcome in NDMM. Further regulatory discussion is also warranted regarding the validity of MRD as a surrogate endpoint for overall survival.

This NMA has some limitations. Given the expected clinical differences between TE and TI populations, indirect comparisons across these settings need to be interpreted cautiously. Furthermore, most treatment comparisons were indirect, and the validity of the network estimates therefore depends on the transitivity assumption, which may be weakened by differences between TE and TI populations in age, fitness, comorbidity, cytogenetic risk, and treatment intensity. MRD assessment was heterogeneous across trials with respect to methodology, sensitivity threshold, timing, analysis population, and outcome definition; in particular, assessments performed after induction, after consolidation, before maintenance, or cumulatively during continuous treatment may not be directly comparable. Lastly, daratumumab- and isatuximab-containing regimens were grouped within common anti-CD38 nodes in the primary analysis, assuming a class effect. When the two antibodies were modeled as separate nodes, daratumumab–VRd + ASCT, daratumumab–KRd and isatuximab–KRd + ASCT ranked highest. These results were consistent with the primary analysis, although differences between nodes of the same backbone partly reflected different MRD assessment timepoints (e.g., GMMG-HD7 assessed MRD before ASCT) rather than differences between antibodies, which cannot be established from indirect comparisons.

Several nodes were informed by a single trial (e.g., anti-CD38–KRd + ASCT, KCd, VMP → Rd), and the power to detect inconsistency was limited; EMN20 had zero events in the Rd arm, requiring a continuity correction. Data for COBRA, ADVANCE, and AGMT-MM04 were derived from conference abstracts, and AGMT-MM04 reported an interim per-protocol analysis.

## 5. Conclusions

In this NMA, anti-CD38-containing quadruplet regimens showed the highest probability of achieving MRD negativity in NDMM, and the addition of an anti-CD38 antibody improved its persistence in both TE and TI populations. The three top-ranked strategies could not be distinguished from one another, and no comparison between strategies with and without ASCT reached statistical significance. The incremental contribution of upfront ASCT in the quadruplet era therefore remains uncertain; direct randomized comparisons with standardized MRD assessment, sustained MRD and long-term outcomes are needed.

## Figures and Tables

**Figure 1:**
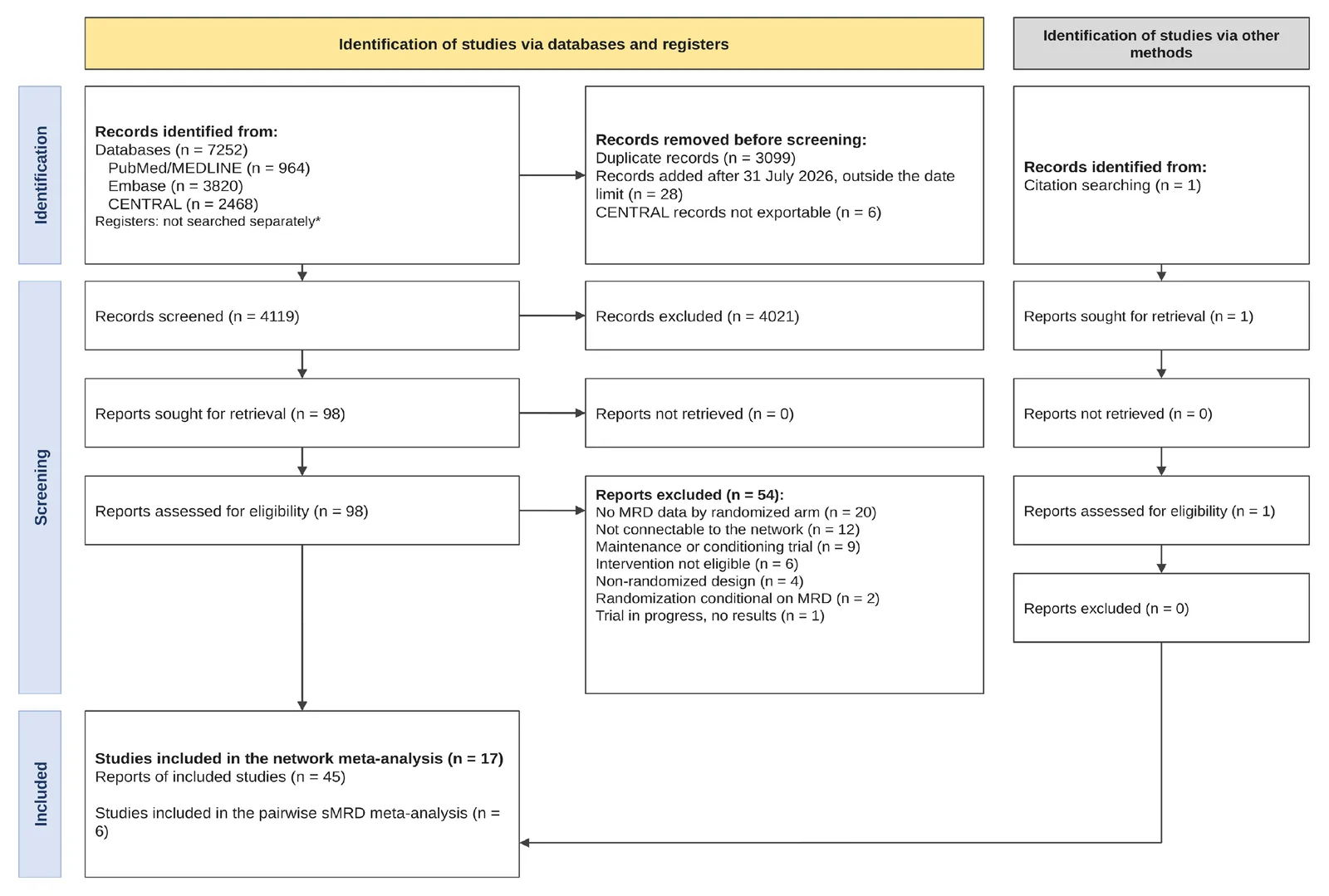
PRISMA for systematic review. * Trial registry records (ClinicalTrials.gov, ICTRP) were retrieved through CENTRAL and screened together with the database records.

**Figure 2:**
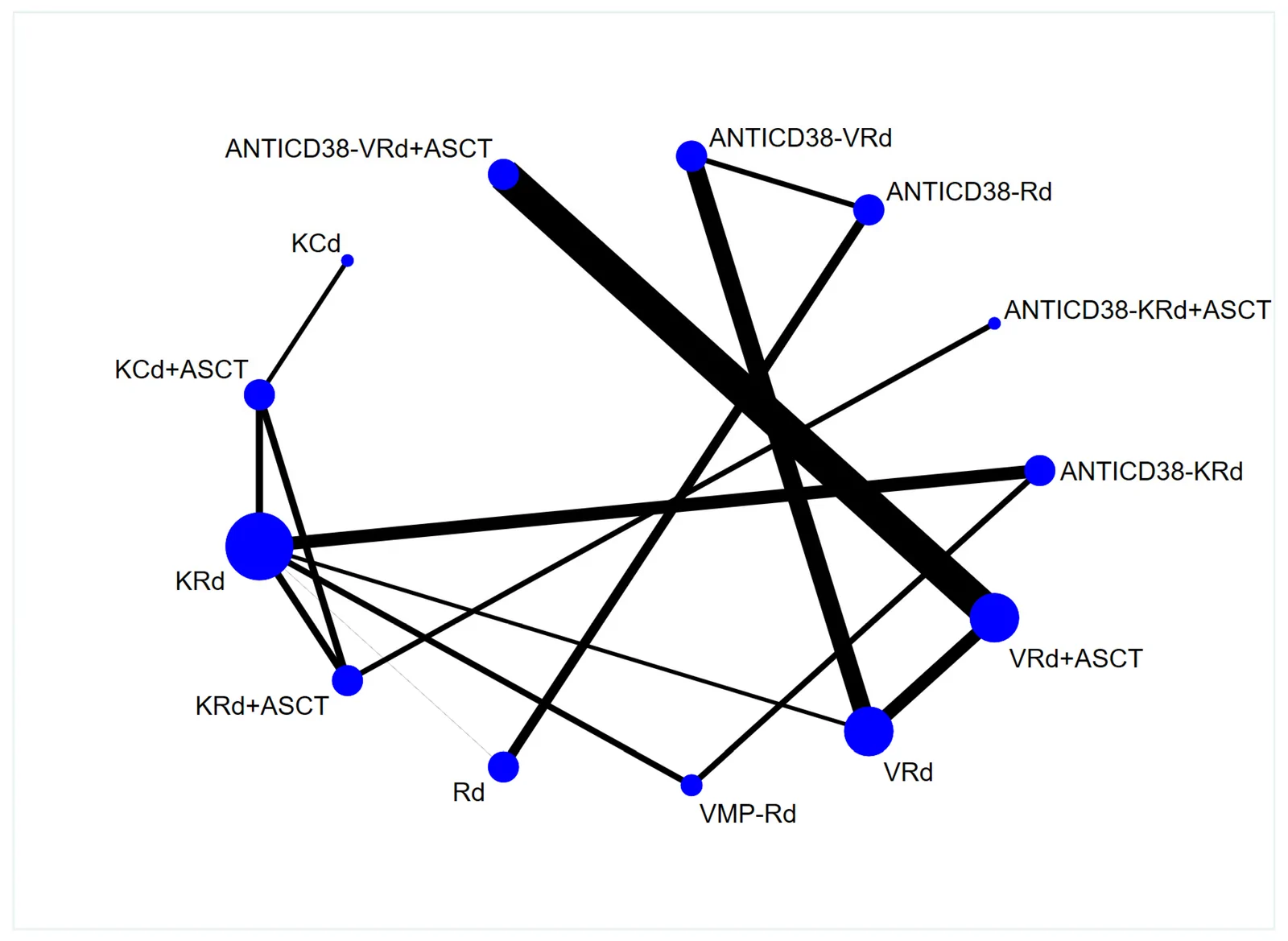
Network of direct comparisons for MRD negativity (17 trials, 13 treatment strategies). Each node represents a treatment strategy, and node size is proportional to the number of trials investigating that strategy. Edges represent direct randomized comparisons; edge thickness is proportional to the inverse variance of the direct estimate. Trials contributing to each edge: anti-CD38–VRd + ASCT vs. VRd + ASCT, PERSEUS, GMMG-HD7 and GRIFFIN; anti-CD38–KRd + ASCT vs. KRd + ASCT, IsKia; VRd + ASCT vs. VRd, DETERMINATION and IFM 2009; KRd + ASCT vs. KRd, KRd + ASCT vs. KCd + ASCT and KRd vs. KCd + ASCT, FORTE (three-arm trial); KCd + ASCT vs. KCd, CARDAMON; anti-CD38–VRd vs. VRd, IMROZ and CEPHEUS; anti-CD38–VRd vs. anti-CD38–Rd, BENEFIT; anti-CD38–Rd vs. Rd, MAIA and AGMT-MM04; KRd vs. VRd, COBRA; KRd vs. Rd, EMN20; anti-CD38–KRd vs. KRd, ADVANCE and GEM2017FIT; anti-CD38–KRd vs. VMP → Rd and KRd vs. VMP → Rd, GEM2017FIT (three-arm trial). ASCT, autologous stem-cell transplantation; C, cyclophosphamide; d, dexamethasone; K, carfilzomib; M, melphalan; P, prednisone; R, lenalidomide; V, bortezomib; VMP → Rd, bortezomib–melphalan–prednisone followed by lenalidomide–dexamethasone.

**Figure 3:**
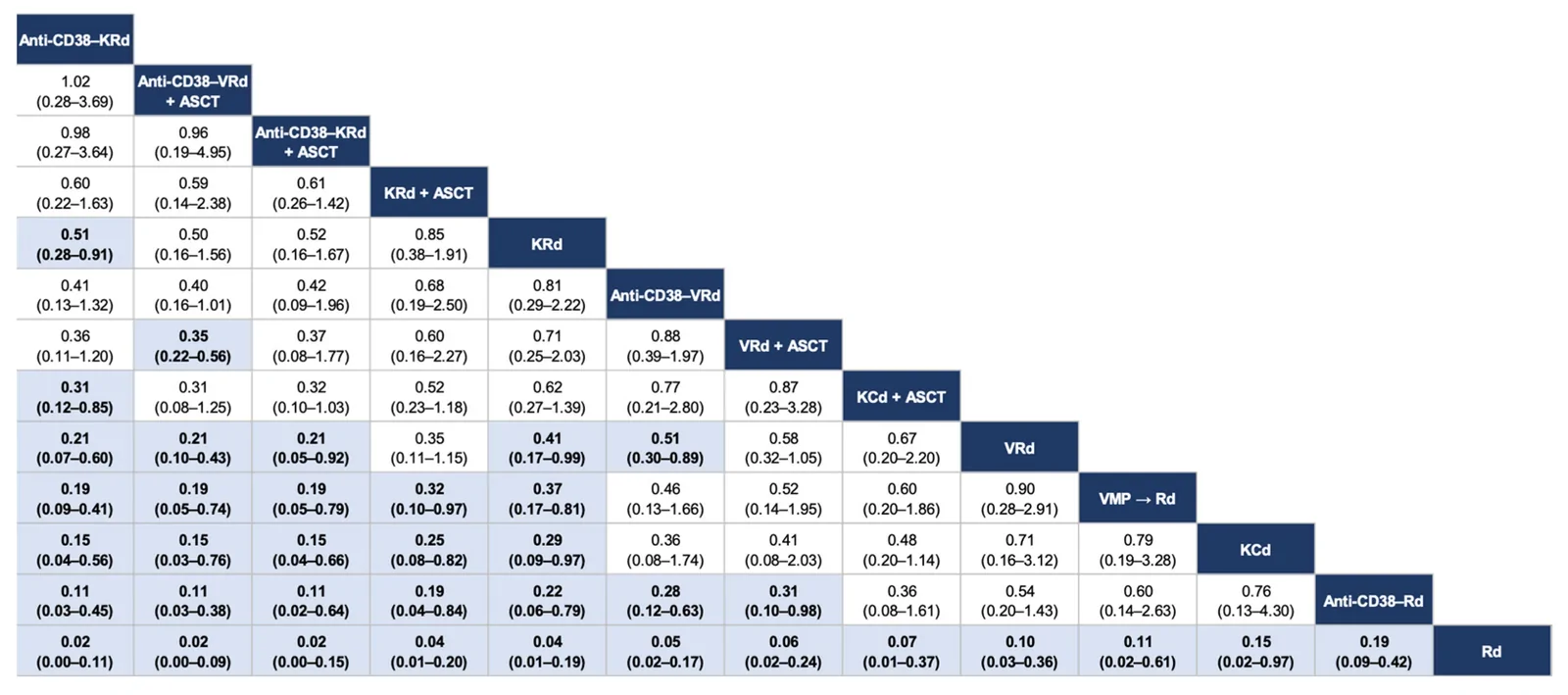
League table of network estimates for MRD negativity (random-effects model). Treatments are ordered from best to worst according to SUCRA values. Each cell reports the odds ratio (OR) with 95% confidence interval (CI) for the row-defining treatment compared with the column-defining treatment; OR < 1 favors the column-defining treatment (i.e., the higher-ranked strategy). Bold, shaded cells indicate comparisons whose 95% CI excludes 1 (statistically significant). ASCT, autologous stem-cell transplantation; C, cyclophosphamide; d, dexamethasone; K, carfilzomib; M, melphalan; P, prednisone; R, lenalidomide; V, bortezomib.

**Figure 4:**
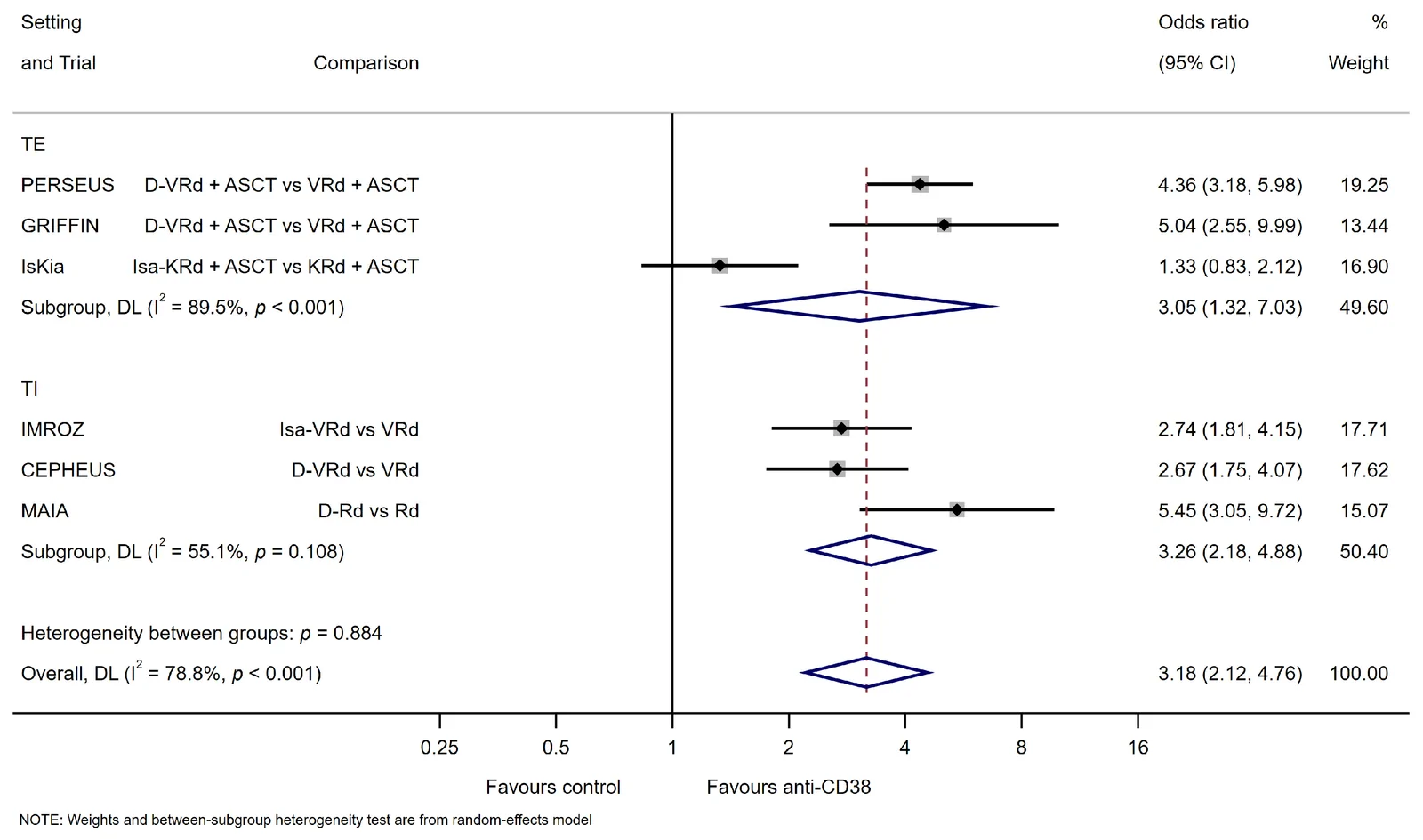
Pairwise meta-analysis of the effect of adding an anti-CD38 antibody on 1-year sustained MRD negativity (10^−5^), by transplant eligibility. Odds ratios > 1 favor the anti-CD38-containing regimen. TE, transplant-eligible; TI, transplant-ineligible. Squares indicate trial-level ORs and horizontal lines their 95% CIs; blue-outlined diamonds indicate pooled subgroup and overall estimates; the dashed vertical line marks the overall pooled OR. DL, DerSimonian–Laird random-effects model.

**Table 1:** Characteristics and measurable residual disease outcomes of the included randomized trials.

Study	Control Arm	Experimental Arm	MRD Assessment	MRD Negativity *	Sustained MRD Negativity *
**ADVANCE**	KRd (triplet) *n* = 139	D-KRd (quadruplet) *n* = 148	Bone-marrow NGS Sensitivity: 10^−5^ Timing: after cycle 8	58.8% vs. 33.1%	Not reported
**AGMT-MM04**	Rd (doublet) *n* = 40 (MRD tested)	Isa-Rd (triplet) *n* = 48 (MRD tested)	Bone-marrow NGF Primary threshold: 10^−6^ Also analyzed at 10^−5^ Timing: end of induction (8 cycles) Per-protocol, interim analysis	10^−5^: 39.6% vs. 2.5% 10^−6^: 35.4% vs. 2.5%	Not reported
**BENEFIT**	Isa-Rd (triplet) *n* = 135	Isa-VRd (quadruplet) *n* = 135	Bone-marrow NGS Primary threshold: 10^−5^ Assay sensitivity: 10^−6^ Flow cytometry at 10^−5^ if NGS unsuccessful Timing: 18 months	52.6% vs. 25.9%	Not reported
**CARDAMON**	KCd induction, then KCd consolidation without ASCT (triplet) *n* = 109	KCd induction, then ASCT (triplet plus ASCT) *n* = 109	Bone-marrow multiparameter flow cytometry Sensitivity: 10^−5^ Timing: 100 days after ASCT or end of KCd consolidation	47.7% vs. 30.3%	Not reported
**CEPHEUS**	VRd followed by Rd (triplet followed by doublet) *n* = 198	D-VRd followed by D-Rd (quadruplet followed by triplet) *n* = 197	Bone-marrow NGS Sensitivity: 10^−5^ Timing: overall (any time)	60.9% vs. 39.4%	≥12 months: 48.7% vs. 26.3%
**COBRA**	VRd (triplet) *n* = 124	KRd (triplet) *n* = 126	Centralized bone-marrow NGS (clonoSEQ) Sensitivity: 10^−5^ and 10^−6^ Timing: 12 months	MRD-negative CR 10^−5^: 31.0% vs. 17.7% 10^−6^: 19.0% vs. 7.3%	Not reported
**DETERMINATION**	VRd without upfront ASCT (triplet) *n* = 357 (MRD evaluated: 108)	VRd plus ASCT (triplet plus ASCT) *n* = 365 (MRD evaluated: 90)	Bone-marrow NGS Sensitivity: 10^−5^ Timing: preliminary analysis in evaluated patients	54.4% vs. 39.8%	Not reported
**EMN20**	Rd (doublet) *n* = 40	KRd (triplet) *n* = 42	Bone-marrow NGS Sensitivity: 10^−5^ Timing: after 2 years of treatment	59.5% vs. 0%	Not reported
**FORTE**	KRd, 12 cycles without ASCT (triplet), *n* = 157 KCd plus ASCT (triplet plus ASCT), *n* = 159	KRd plus ASCT (triplet plus ASCT) *n* = 158	Eight-color multiparameter flow cytometry Approximate sensitivity: 10^−5^ Timing: premaintenance	KRd plus ASCT: 58% KRd12: 54% KCd plus ASCT: 42%	1 year: 47%, 35%, and 25%, respectively
**GEM2017FIT**	VMP followed by Rd (sequential), *n* = 154	KRd (triplet), *n* = 154 D-KRd (quadruplet), *n* = 153	Bone-marrow NGF Sensitivity: 10^−5^ Timing: after 18 cycles	D-KRd: 61.4% KRd: 53.9% VMP-Rd: 26.6%	Not reported
**GMMG-HD7**	VRd (triplet) *n* = 329	Isa-VRd (quadruplet) *n* = 331	Bone-marrow NGF Sensitivity: 10^−5^ Timing: end of induction (before ASCT)	50.1% vs. 35.6%	Not reported
**GRIFFIN**	VRd plus ASCT, then R maintenance (triplet plus ASCT) *n* = 103	D-VRd plus ASCT, then D-R maintenance (quadruplet plus ASCT) *n* = 104	Bone-marrow NGS Sensitivity: 10^−5^ Timing: end of maintenance (cumulative)	64.4% vs. 30.1%	≥12 months: 44.2% vs. 13.6%
**IFM 2009**	VRd without upfront ASCT (triplet) *n* = 350 (MRD evaluated: 264)	VRd plus ASCT (triplet plus ASCT) *n* = 350 (MRD evaluated: 245)	Bone-marrow NGS/deep sequencing Sensitivity: 10^−6^ Timing: at least once during maintenance	29.8% vs. 20.5% at 10^−6^	Not reported
**IMROZ**	VRd followed by Rd (triplet followed by doublet) *n* = 181	Isa-VRd followed by Isa-Rd (quadruplet followed by triplet) *n* = 265	Bone-marrow NGS Sensitivity: 10^−5^ Timing: overall (any time)	58.1% vs. 43.6%	≥12 months: 46.8% vs. 24.3%
**IsKia**	KRd plus ASCT (triplet plus ASCT) *n* = 151	Isa-KRd plus ASCT (quadruplet plus ASCT) *n* = 151	Bone-marrow NGS Primary threshold: 10^−5^ Also assessed at 10^−6^ Parallel flow-cytometry evaluation Timing: post-consolidation	10^−5^: 76.8% vs. 66.9% 10^−6^: 68% vs. 48%	1 year at 10^−5^: 66% vs. 59% 1 year at 10^−6^: 52% vs. 38%
**MAIA**	Rd (doublet) *n* = 369	D-Rd (triplet) *n* = 368	Bone-marrow NGS (clonoSEQ) Sensitivity: 10^−5^ Timing: overall (any time)	32.1% vs. 11.1%	≥12 months: 18.8% vs. 4.1% ≥18 months: 16.8% vs. 3.3%
**PERSEUS**	VRd plus ASCT, then lenalidomide (triplet plus ASCT) *n* = 354	D-VRd plus ASCT, then D-R maintenance (quadruplet plus ASCT) *n* = 355	Bone-marrow NGS (clonoSEQ) Sensitivity: 10^−5^ and 10^−6^ Timing: overall (any time)	10^−5^: 75.2% vs. 47.5% 10^−6^: 65.1% vs. 32.2%	≥12 months at 10^−5^: 64.8% vs. 29.7% ≥24 months: 55.8% vs. 22.6%

* Unless otherwise specified, MRD outcomes are reported as experimental versus control. For FORTE, percentages are presented in the order specified within each cell. Abbreviations: ASCT, autologous stem-cell transplantation; C, cyclophosphamide; CR, complete response; D, daratumumab; d, dexamethasone; Isa, isatuximab; K, carfilzomib; MRD, measurable residual disease; NGF, next-generation flow; NGS, next-generation sequencing; R, lenalidomide; V, bortezomib.

**Table 2:** Treatment strategies (network nodes) included in the network meta-analysis.

Strategy (Network Node)	Components	Setting	Trials in the Network
**Non-transplant strategies**			
**VRd**	Bortezomib (proteasome inhibitor), lenalidomide (immunomodulatory drug), dexamethasone; in TI trials followed by Rd until progression; in TE trials with ASCT deferred, followed by lenalidomide maintenance	TI; TE with ASCT deferred	IMROZ, CEPHEUS, DETERMINATION, IFM 2009, COBRA
**Anti-CD38–VRd**	Daratumumab (D-VRd) or isatuximab (Isa-VRd) plus VRd, followed by anti-CD38–Rd	TI; TE with ASCT deferred	IMROZ, CEPHEUS, BENEFIT
**Rd**	Lenalidomide and dexamethasone, continuous until progression	TI	MAIA, EMN20, AGMT-MM04
**Anti-CD38–Rd**	Daratumumab (D-Rd) or isatuximab (Isa-Rd) plus Rd	TI	MAIA, BENEFIT, AGMT-MM04
**KRd**	Carfilzomib (proteasome inhibitor), lenalidomide, dexamethasone, without upfront ASCT	TE with ASCT deferred; TI (fit)	COBRA, FORTE, ADVANCE, EMN20, GEM2017FIT
**Anti-CD38–KRd**	Daratumumab plus KRd (D-KRd)	TE with ASCT deferred; TI (fit)	ADVANCE, GEM2017FIT
**KCd**	Carfilzomib, cyclophosphamide, dexamethasone induction and consolidation without ASCT, followed by carfilzomib maintenance	TE	CARDAMON
**VMP → Rd**	Bortezomib, melphalan, prednisone (9 cycles) followed by Rd (9 cycles)	TI (fit, 65–80 years)	GEM2017FIT
**Transplant-containing strategies**			
**VRd + ASCT**	VRd induction, high-dose melphalan and ASCT, consolidation and/or lenalidomide maintenance	TE	PERSEUS, GMMG-HD7, GRIFFIN, DETERMINATION, IFM 2009
**Anti-CD38–VRd + ASCT**	Daratumumab (D-VRd) or isatuximab (Isa-VRd) plus VRd induction, ASCT, consolidation and maintenance (with anti-CD38 in PERSEUS and GRIFFIN)	TE	PERSEUS, GMMG-HD7, GRIFFIN
**KRd + ASCT**	KRd induction, ASCT and KRd consolidation	TE	FORTE, IsKia
**Anti-CD38–KRd + ASCT**	Isatuximab plus KRd (Isa-KRd) induction, ASCT and consolidation	TE	IsKia
**KCd + ASCT**	KCd induction and ASCT, followed by KCd consolidation (FORTE) or carfilzomib maintenance (CARDAMON)	TE	FORTE, CARDAMON

Maintenance and consolidation schedules varied across trials and are detailed in Table 1. ASCT, autologous stem-cell transplantation (high-dose melphalan); C, cyclophosphamide; D, daratumumab; d, dexamethasone; Isa, isatuximab; K, carfilzomib; M, melphalan; P, prednisone; R, lenalidomide; TE, transplant-eligible; TI, transplant-ineligible; V, bortezomib.

**Table 3:** Summary of SUCRA and PrBest values.

Rank	Treatment	SUCRA (%)	PrBest (%)
1	Anti-CD38–KRd	89.1	27.6
2	Anti-CD38–VRd + ASCT	88.0	36.9
3	Anti-CD38–KRd + ASCT	86.5	34.1
4	KRd + ASCT	70.8	0.9
5	KRd	65.3	0.0
6	Anti-CD38–VRd	57.4	0.4
7	VRd + ASCT	51.9	0.0
8	KCd + ASCT	45.6	0.1
9	VRd	29.1	0.0
10	VMP → Rd	27.9	0.0
11	KCd	22.2	0.0
12	Anti-CD38–Rd	15.8	0.0
13	Rd	0.3	0.0

## Data Availability

The data presented in this study are available within the article and its Supplementary Materials.

## Supplementary Materials

The following supporting information can be downloaded at https://www.mdpi.com/article/10.3390/cancers18213573/s1, Supplementary S1: PRISMA-NMA checklist; Supplementary S2: Search strategies for PubMed/MEDLINE, Embase and CENTRAL; Supplementary S3: Stata code used for all analyses; Supplementary S4: Assignment of randomized arms to network nodes; Supplementary S5: Sensitivity analyses; Supplementary S6: Risk-of-bias assessment of the included trials; Supplementary S7: CINeMA assessment of confidence in the evidence.
