# Supplementary material for: Achievement and Persistence of MRD Negativity with Anti-CD38-Based Regimens in Newly Diagnosed Multiple Myeloma: A Systematic Review and Network Meta-Analysis
Source: Cancers (Basel). 2026 Nov 6;18(21):3573. doi: 10.3390/cancers18213573 (PMC13761838; doi:10.3390/cancers18213573)
Supplement: Supplementary file 1 [file cancers-18-03573-s001.zip › cancers-4598515-supplementary.pdf]

Supplementary S1 – PRISMA-NMA Checklist

Section/Topic	Item #	Checklist Item	Reported In
**TITLE**			
Title	1	Identify the report as a systematic review *incorporating a network meta-analysis (or related form of meta-analysis).*	Title
**ABSTRACT**			
Structured summary	2	Provide a structured summary including, as applicable:<br>**Background:** main objectives<br>**Methods:** data sources; study eligibility criteria, participants, and interventions; study appraisal; and *synthesis methods, such as network meta-analysis.*<br>**Results:** number of studies and participants identified; summary estimates with corresponding confidence/credible intervals; *treatment rankings may also be discussed. Authors may choose to summarize pairwise comparisons against a chosen treatment included in their analyses for brevity.*<br>**Discussion/Conclusions:** limitations; conclusions and implications of findings.<br>**Other:** primary source of funding; systematic review registration number with registry name.	Abstract
**INTRODUCTION**			
Rationale	3	Describe the rationale for the review in the context of what is already known*, including mention of why a network meta-analysis has been conducted.*	Introduction
Objectives	4	Provide an explicit statement of questions being addressed, with reference to participants, interventions, comparisons, outcomes, and study design (PICOS).	Introduction
**METHODS**			
Protocol and registration	5	Indicate whether a review protocol exists and if and where it can be accessed (e.g., Web address); and, if available, provide registration information, including registration number.	Methods
Eligibility criteria	6	Specify study characteristics (e.g., PICOS, length of follow-up) and report characteristics (e.g., years considered, language, publication status) used as criteria for eligibility, giving rationale. *Clearly describe eligible treatments included in the treatment network, and note whether any have been clustered or merged into the same node (with justification).*	Methods; Table 2
Information sources	7	Describe all information sources (e.g., databases with dates of coverage, contact with study authors to identify additional studies) in the search and date last searched.	Methods
Search	8	Present full electronic search strategy for at least one database, including any limits used, such that it could be repeated.	Suppl. S2
Study selection	9	State the process for selecting studies (i.e., screening, eligibility, included in systematic review, and, if applicable,	Methods; Fig. 1

		included in the meta-analysis).	
Data collection process	10	Describe method of data extraction from reports (e.g., piloted forms, independently, in duplicate) and any processes for obtaining and confirming data from investigators.	Methods
Data items	11	List and define all variables for which data were sought (e.g., PICOS, funding sources) and any assumptions and simplifications made.	Methods; Table 1
**Geometry of the network**	**S2**	Describe methods used to explore the geometry of the treatment network under study and potential biases related to it. This should include how the evidence base has been graphically summarized for presentation, and what characteristics were compiled and used to describe the evidence base to readers.	Methods; Results 3.3
Risk of bias within individual studies	12	Describe methods used for assessing risk of bias of individual studies (including specification of whether this was done at the study or outcome level), and how this information is to be used in any data synthesis.	Methods; Suppl. S6
Summary measures	13	State the principal summary measures (e.g., risk ratio, difference in means). *Also describe the use of additional summary measures assessed, such as treatment rankings and surface under the cumulative ranking curve (SUCRA) values, as well as modified approaches used to present summary findings from meta-analyses.*	Methods
Planned methods of analysis	14	Describe the methods of handling data and combining results of studies for each network meta-analysis. This should include, but not be limited to:<br>• *Handling of multi-arm trials;*<br>• *Selection of variance structure;*<br>• *Selection of prior distributions in Bayesian analyses; and*<br>• *Assessment of model fit.*	Methods; Suppl. S3
**Assessment of Inconsistency**	**S4**	Describe the statistical methods used to evaluate the agreement of direct and indirect evidence in the treatment network(s) studied. Describe efforts taken to address its presence when found.	Methods
Risk of bias across studies	15	Specify any assessment of risk of bias that may affect the cumulative evidence (e.g., publication bias, selective reporting within studies).	Methods
Additional analyses	16	Describe methods of additional analyses if done, indicating which were pre-specified. This may include, but not be limited to, the following:<br>• Sensitivity or subgroup analyses;<br>• Meta-regression analyses;<br>• *Alternative formulations of the treatment network; and*<br>• *Use of alternative prior distributions for Bayesian analyses (if applicable).*	Methods

RESULTS†

Study selection	17	Give numbers of studies screened, assessed for eligibility, and included in the review, with reasons for exclusions at each stage, ideally with a flow diagram.	Results 3.1; Fig. 1
**Presentation of network structure**	**S5**	Provide a network graph of the included studies to enable visualization of the geometry of the treatment network.	Fig. 2
**Summary of network geometry**	**S6**	Provide a brief overview of characteristics of the treatment network. This may include commentary on the abundance of trials and randomized patients for the different interventions and pairwise comparisons in the network, gaps of evidence in the treatment network, and potential biases reflected by the network structure.	Results 3.3
Study characteristics	18	For each study, present characteristics for which data were extracted (e.g., study size, PICOS, follow-up period) and provide the citations.	Tables 1–2; Suppl. S4
Risk of bias within studies	19	Present data on risk of bias of each study and, if available, any outcome level assessment.	Results 3.2; Suppl. S6
Results of individual studies	20	For all outcomes considered (benefits or harms), present, for each study: 1) simple summary data for each intervention group, and 2) effect estimates and confidence intervals. *Modified approaches may be needed to deal with information from larger networks.*	Table 1; Suppl. S4
Synthesis of results	21	Present results of each meta-analysis done, including confidence/credible intervals. *In larger networks, authors may focus on comparisons versus a particular comparator (e.g. placebo or standard care), with full findings presented in an appendix. League tables and forest plots may be considered to summarize pairwise comparisons.* If additional summary measures were explored (such as treatment rankings), these should also be presented.	Results 3.4, 3.6; Table 3; Figs. 3–4
**Exploration for inconsistency**	**S7**	Describe results from investigations of inconsistency. This may include such information as measures of model fit to compare consistency and inconsistency models, *P* values from statistical tests, or summary of inconsistency estimates from different parts of the treatment network.	Results 3.5
Risk of bias across studies	22	Present results of any assessment of risk of bias across studies for the evidence base being studied.	Results 3.7; Suppl. S7
Results of additional analyses	23	Give results of additional analyses, if done (e.g., sensitivity or subgroup analyses, meta-regression analyses, *alternative network geometries studied, alternative choice of prior distributions for Bayesian analyses,* and so forth).	Results 3.5, 3.7; Suppl. S5, S7
**DISCUSSION**			
Summary of evidence	24	Summarize the main findings, including the strength of evidence for each main outcome; consider their relevance to key groups (e.g., healthcare providers, users, and policy-makers).	Discussion
Limitations	25	Discuss limitations at study and outcome level (e.g., risk of bias), and at review level (e.g., incomplete retrieval of identified research, reporting bias). *Comment on the validity of the assumptions, such as transitivity and consistency. Comment on any concerns regarding network geometry (e.g., avoidance of certain comparisons).*	Discussion
Conclusions	26	Provide a general interpretation of the results in the context of other evidence, and implications for future research.	Conclusions

**FUNDING**			
Funding	27	Describe sources of funding for the systematic review and other support (e.g., supply of data); role of funders for the systematic review. This should also include information regarding whether funding has been received from manufacturers of treatments in the network and/or whether some of the authors are content experts with professional conflicts of interest that could affect use of treatments in the network.	Funding

Supplementary S2 – Queries

PubMed query

(("Multiple Myeloma"[Mesh] OR myeloma[tiab] OR "plasma cell myeloma"[tiab]) AND ("newly diagnosed"[tiab] OR NDMM[tiab] OR untreated[tiab] OR "previously untreated"[tiab] OR frontline[tiab] OR "front-line"[tiab] OR "first-line"[tiab] OR "first line"[tiab] OR upfront[tiab] OR "up-front"[tiab] OR induction[tiab] OR "transplant-eligible"[tiab] OR "transplant eligible"[tiab] OR "transplant-ineligible"[tiab] OR "transplant ineligible"[tiab]) AND (daratumumab[tiab] OR isatuximab[tiab] OR "anti-CD38"[tiab] OR bortezomib[tiab] OR carfilzomib[tiab] OR lenalidomide[tiab] OR "Bortezomib"[Mesh] OR "Lenalidomide"[Mesh] OR "Transplantation, Autologous"[Mesh] OR "Hematopoietic Stem Cell Transplantation"[Mesh] OR "autologous stem cell transplantation"[tiab] OR "autologous transplant*"[tiab] OR ASCT[tiab]) AND ((randomized controlled trial[pt] OR controlled clinical trial[pt] OR randomized[tiab] OR randomised[tiab] OR placebo[tiab] OR "clinical trials as topic"[mesh:noexp] OR randomly[tiab] OR trial[ti]) NOT (animals[mh] NOT humans[mh]))) AND ("1900/01/01"[dp] : "2026/07/31"[dp])

Embase query

('multiple myeloma'/exp OR myeloma:ti,ab OR 'plasma cell myeloma':ti,ab) AND ('newly diagnosed':ti,ab OR ndmm:ti,ab OR untreated:ti,ab OR 'previously untreated':ti,ab OR frontline:ti,ab OR 'front-line':ti,ab OR 'first-line':ti,ab OR 'first line':ti,ab OR upfront:ti,ab OR 'up-front':ti,ab OR induction:ti,ab OR 'transplant eligible':ti,ab OR 'transplant-eligible':ti,ab OR 'transplant ineligible':ti,ab OR 'transplant-ineligible':ti,ab) AND ('daratumumab'/exp OR 'isatuximab'/exp OR 'bortezomib'/exp OR 'carfilzomib'/exp OR 'lenalidomide'/exp OR 'autologous stem cell transplantation'/exp OR daratumumab:ti,ab OR isatuximab:ti,ab OR 'anti-cd38':ti,ab OR bortezomib:ti,ab OR carfilzomib:ti,ab OR lenalidomide:ti,ab OR 'autologous stem cell transplantation':ti,ab OR 'autologous transplant*':ti,ab OR asct:ti,ab) AND ('randomized controlled trial'/exp OR 'controlled clinical trial'/exp OR random*:ti,ab OR placebo:ti,ab OR 'double blind procedure'/de OR 'single blind procedure'/de OR 'crossover procedure'/de OR trial:ti) NOT ('animal'/exp NOT 'human'/exp)

CENTRAL query

([mh "Multiple Myeloma"] OR (myeloma OR "plasma cell myeloma"):ti,ab,kw) AND (("newly diagnosed" OR NDMM OR untreated OR "previously untreated" OR frontline OR "front-line" OR "first-line" OR "first line" OR upfront OR "up-front" OR induction OR "transplant eligible" OR "transplant-eligible" OR "transplant ineligible" OR "transplant-ineligible"):ti,ab,kw) AND ((daratumumab OR isatuximab OR "anti-CD38" OR bortezomib OR carfilzomib OR lenalidomide OR "autologous stem cell transplantation" OR (autologous NEXT transplant*) OR ASCT):ti,ab,kw OR [mh Bortezomib] OR [mh Lenalidomide] OR [mh "Transplantation, Autologous"])

Supplementary S3 – Stata code

S3.1 – Functions (ranking, SUCRA and league table)

```
mata:
mata clear

void nma_read(string scalar trtlist, beta, S, names)
{
    names = tokens(trtlist)
    K = cols(names)
    b = st_matrix("e(b)"); V = st_matrix("e(V)")
    eqs = st_matrixcolstripe("e(b)")[.,1]
    beta = J(K,1,0); S = J(K,K,0)
    sel = J(0,1,.); code = J(0,1,.)
    for (i=1; i<=rows(eqs); i++) {
        if (strpos(eqs[i],"_y_")==1) {
            s = subinstr(eqs[i],"_y_","")
            c = strtoreal(s)
            if (c>=.) c = ascii(s)-64
            sel = sel \ i ; code = code \ c
        }
    }
    if (rows(sel)!=K-1) printf("{err}ATTENZIONE: letti %f coefficienti, attesi %f\n", rows(sel), K-1)
    for (i=1; i<=rows(sel); i++) {
        beta[code[i]] = b[sel[i]]
        for (j=1; j<=rows(sel); j++) S[code[i],code[j]] = V[sel[i],sel[j]]
    }
}

void nma_rank(beta, S, sucra, pbest, mr)
{
    K = rows(beta); reps = 5000
    nz = select((1::K), diagonal(S):>0)
    L = cholesky(S[nz,nz])
    D = J(reps, K, 0)
    D[., nz'] = J(reps,1,beta[nz]') + rnormal(reps, rows(nz), 0, 1)*L'
    R = J(reps, K, .)
    for (r=1; r<=reps; r++) {
        o = order(-D[r,.]', 1)
        R[r, o'] = (1..K)
    }
    mr = mean(R)
    sucra = (K :- mr) :/ (K-1) :* 100
    pbest = mean(R:==1) :* 100
}

void nma_summ(string scalar lab, string scalar trtlist, string scalar outfile)
{
    beta = .; S = .; names = ""
    nma_read(trtlist, beta, S, names)
    K = cols(names)
    sucra = .; pbest = .; mr = .
    nma_rank(beta, S, sucra, pbest, mr)
    Sig = st_matrix("e(Sigma)")
    tau = (rows(Sig)>0 ? sqrt(Sig[1,1]) : 0)
    ordr = order(-sucra', 1)
    printf("\n{res}=== %s ===   tau = %5.3f\n", lab, tau)
    printf("{txt}%-20s %8s %16s %7s %7s %8s\n", "Treatment", "OR vsREF", "95% CI", "SUCRA", "PrBest", 
"MeanRank")
    if (outfile!="") fh = fopen(outfile, "a")
    for (k=1; k<=K; k++) {
        i = ordr[k]; se = sqrt(S[i,i])
        orr = exp(beta[i]); lo = exp(beta[i]-1.96*se); hi = exp(beta[i]+1.96*se)
        if (se==0) printf("%-20s %8s %16s %7.1f %7.1f %8.1f\n", names[i], "ref", "", sucra[i], pbest[i], 
mr[i])
        else printf("%-20s %8.2f   (%5.2f-%6.2f) %7.1f %7.1f %8.1f\n", names[i], orr, lo, hi, sucra[i], 
pbest[i], mr[i])
        if (outfile!="") {
            if (se==0) fput(fh, sprintf("%s,%s,1,,,%5.1f,%5.1f,%4.1f,%5.3f", lab, names[i], sucra[i], 
pbest[i], mr[i], tau))
            else fput(fh, sprintf("%s,%s,%5.3f,%5.3f,%5.3f,%5.1f,%5.1f,%4.1f,%5.3f", lab, names[i], orr, 
lo, hi, sucra[i], pbest[i], mr[i], tau))
        }
    }
    if (outfile!="") fclose(fh)
}

void nma_league(string scalar trtlist, string scalar xlsfile)
{
    class xl scalar X
```

```
    beta = .; S = .; names = ""
    nma_read(trtlist, beta, S, names)
    K = cols(names)
    sucra = .; pbest = .; mr = .
    nma_rank(beta, S, sucra, pbest, mr)
    ord = order(-sucra', 1)'
    M = J(K,K,"")
    for (r=1; r<=K; r++) {
        for (c=1; c<=K; c++) {
            i = ord[r]; j = ord[c]
            if (r==c) M[r,c] = names[i]
            else {
                d = beta[i]-beta[j]
                se = sqrt(S[i,i]+S[j,j]-2*S[i,j])
                M[r,c] = sprintf("%4.2f (%4.2f-%4.2f)", exp(d), exp(d-1.96*se), exp(d+1.96*se))
            }
        }
    }
    M
    X = xl()
    X.create_book(xlsfile, "League MRD", "xlsx")
    X.put_string(1, 1, M)
    X.close_book()
}
end

capture program drop nmarun
program define nmarun
    syntax , LABel(string) [FIXED OUTfile(string)]
    quietly levelsof TRT, local(trts) clean
    quietly network setup d n, studyvar(ID) trtvar(TRT) ref(VRd) numcodes
    network meta consistency, `fixed'
    mata: nma_summ(`"`label'"', "`trts'", `"`outfile'"')
end
```

S3.2 – Primary network meta-analysis

```
set seed 20261024
keep TrialsAuthors ID SETTING TRT PATIENT MRDneg
drop if missing(ID)
rename MRDneg d
rename PATIENT n
replace TrialsAuthors = strtrim(TrialsAuthors)
replace TRT = strtrim(TRT)
duplicates drop ID TRT d n, force

duplicates report ID TRT
tab TrialsAuthors
tab TRT
quietly summarize n
display "Totale pazienti: " r(sum)
quietly levelsof TRT, local(trts) clean

network setup d n, studyvar(ID) trtvar(TRT) ref(VRd) numcodes
network table

network convert pairs
gen invvarES = 1/(_stderr^2)
networkplot _t1 _t2, edgew(invvarES) edgesc(1.2) asp(0.8) lab(`trts')
network convert augment
capture drop _t1 _t2 invvarES

network meta consistency, fixed
network meta inconsistency
capture noisily network sidesplit all
network meta consistency

mata: nma_summ("Primary (random effects)", "`trts'", "")

quietly network meta consistency
mata: nma_league("`trts'", "League_MRD_v3.xlsx")
```

S3.3 – Sensitivity analyses

```
set seed 20261024
keep TrialsAuthors ID SETTING TRT PATIENT MRDneg
drop if missing(ID)
rename MRDneg d
rename PATIENT n
replace TrialsAuthors = strtrim(TrialsAuthors)
replace TRT = strtrim(TRT)
```

```
duplicates drop ID TRT d n, force

global OUTFILE "Sensitivity_MRD_v3.csv"
capture file close fh
file open fh using "$OUTFILE", write replace
file write fh "analysis,treatment,OR_vs_VRd,lower95,upper95,SUCRA,PrBest,MeanRank,tau" _n
file close fh

preserve
nmarun, label("Primary (random effects)") outfile("$OUTFILE")
restore

preserve
nmarun, label("Fixed effect") fixed outfile("$OUTFILE")
restore

preserve
drop if TrialsAuthors=="GMMG-HD7"
nmarun, label("Excluding GMMG-HD7") outfile("$OUTFILE")
restore

preserve
drop if TrialsAuthors=="IFM 2009"
nmarun, label("10-5 threshold only") outfile("$OUTFILE")
restore

preserve
drop if inlist(TrialsAuthors,"ADVANCE","COBRA","AGMT-MM04")
nmarun, label("Excluding abstract-only data") outfile("$OUTFILE")
restore

preserve
keep if inlist(TrialsAuthors,"PERSEUS","GMMG-HD7","GRIFFIN","IsKia","DETERMINATION","IFM
2009","FORTE","COBRA","ADVANCE") | TrialsAuthors=="CARDAMON"
nmarun, label("TE/deferred network") outfile("$OUTFILE")
restore

preserve
keep if inlist(TrialsAuthors,"IMROZ","CEPHEUS","MAIA","BENEFIT","EMN20","GEM2017FIT","AGMT-MM04")
nmarun, label("TI network") outfile("$OUTFILE")
restore

preserve
replace TRT="DARA-VRd+ASCT" if inlist(TrialsAuthors,"PERSEUS","GRIFFIN") & TRT=="ANTICD38-VRd+ASCT"
replace TRT="ISA-VRd+ASCT"  if TrialsAuthors=="GMMG-HD7" & TRT=="ANTICD38-VRd+ASCT"
replace TRT="ISA-VRd"       if inlist(TrialsAuthors,"IMROZ","BENEFIT") & TRT=="ANTICD38-VRd"
replace TRT="DARA-VRd"      if TrialsAuthors=="CEPHEUS" & TRT=="ANTICD38-VRd"
replace TRT="ISA-Rd"        if inlist(TrialsAuthors,"BENEFIT","AGMT-MM04") & TRT=="ANTICD38-Rd"
replace TRT="DARA-Rd"       if TrialsAuthors=="MAIA"    & TRT=="ANTICD38-Rd"
replace TRT="ISA-KRd+ASCT"  if TrialsAuthors=="IsKia"   & TRT=="ANTICD38-KRd+ASCT"
replace TRT="DARA-KRd"      if inlist(TrialsAuthors,"ADVANCE","GEM2017FIT") & TRT=="ANTICD38-KRd"
tab TRT
nmarun, label("Separate daratumumab/isatuximab nodes") outfile("$OUTFILE")
restore

foreach t in "PERSEUS" "IMROZ" "MAIA" "CEPHEUS" "GMMG-HD7" "GRIFFIN" "IsKia" "DETERMINATION" "IFM 2009"
"COBRA" "BENEFIT" "ADVANCE" "CARDAMON" "EMN20" "GEM2017FIT" "AGMT-MM04" {
    preserve
    drop if TrialsAuthors=="`t'"
    nmarun, label("LOO - without `t'") outfile("$OUTFILE")
    restore
}

display _n "Risultati salvati in: $OUTFILE"
```

S3.4 – Pairwise meta-analysis of sustained MRD negativity

```
keep Trial Setting Backbone Comparison e1 n1 e0 n0
drop if missing(e1)
gen f1 = n1 - e1
gen f0 = n0 - e0
list Trial Setting e1 n1 e0 n0, noobs sepby(Setting)

metan e1 f1 e0 f0, or random by(Setting) label(namevar=Trial) effect(Odds ratio) lcols(Comparison)
favours(Favours control # Favours anti-CD38) xlabel(0.25 0.5 1 2 4 8 16) force
metan e1 f1 e0 f0, or model(reml) by(Setting) label(namevar=Trial) nograph
metan e1 f1 e0 f0, or fixed by(Setting) label(namevar=Trial) nograph
metan e1 f1 e0 f0, or random label(namevar=Trial) influence nograph
```

Supplementary S4 – Assignment of randomized arms to network nodes

Trial	Randomized arm	Network node	MRD-negative, n	Analysed, n
**ADVANCE**	D-KRd	Anti-CD38–KRd	87	148
	KRd	KRd	46	139
**AGMT-MM04**	Isa-Rd, then Isa-R	Anti-CD38–Rd	19	48
	Rd, then R	Rd	1	40
**BENEFIT**	Isa-VRd	Anti-CD38–VRd	71	135
	Isa-Rd	Anti-CD38–Rd	35	135
**CARDAMON**	KCd induction, then ASCT	KCd + ASCT	52	109
	KCd induction, then KCd consolidation	KCd	33	109
**CEPHEUS**	D-VRd, then D-Rd	Anti-CD38–VRd	120	197
	VRd, then Rd	VRd	78	198
**COBRA**	KRd	KRd	39	126
	VRd	VRd	22	124
**DETERMINATION**	VRd plus ASCT	VRd + ASCT	49	90
	VRd, ASCT deferred	VRd	43	108
**EMN20**	KRd	KRd	25	42
	Rd	Rd	0	40
**FORTE**	KRd plus ASCT	KRd + ASCT	92	158
	KRd, 12 cycles	KRd	85	157
	KCd plus ASCT	KCd + ASCT	67	159
**GEM2017FIT**	D-KRd	Anti-CD38–KRd	94	153
	KRd	KRd	83	154
	VMP ×9, then Rd ×9	VMP → Rd	41	154
**GMMG-HD7**	Isa-VRd, then ASCT	Anti-CD38–VRd + ASCT	166	331
	VRd, then ASCT	VRd + ASCT	117	329
**GRIFFIN**	D-VRd plus ASCT, then D-R	Anti-CD38–VRd + ASCT	67	104
	VRd plus ASCT, then R	VRd + ASCT	31	103
**IFM 2009**	VRd plus ASCT	VRd + ASCT	73	245
	VRd, ASCT deferred	VRd	54	264
**IMROZ**	Isa-VRd, then Isa-Rd	Anti-CD38–VRd	154	265
	VRd, then Rd	VRd	79	181
**IsKia**	Isa-KRd plus ASCT	Anti-CD38–KRd + ASCT	116	151
	KRd plus ASCT	KRd + ASCT	101	151

Trial	Randomized arm	Network node	MRD-negative, n	Analysed, n
**MAIA**	D-Rd	Anti-CD38–Rd	118	368
	Rd	Rd	41	369
**PERSEUS**	D-VRd plus ASCT, then D-R	Anti-CD38–VRd + ASCT	267	355
	VRd plus ASCT, then R	VRd + ASCT	168	354

Supplementary S5 – Sensitivity analysis

Analysis	Trials (patients)	τ	Three top-ranked strategies (SUCRA, %)	VRd+ASCT vs VRd, OR (95% CI)
**Model, population and node definition**				
**Primary analysis (random effects)**	17 (6,293)	0.35	Anti-CD38–KRd (89.1)<br>Anti-CD38–VRd+ASCT (88.0)<br>Anti-CD38–KRd+ASCT (86.5)	1.72 (0.95–3.10)
Fixed-effect model	17 (6,293)	0.00	Anti-CD38–KRd (90.9)<br>Anti-CD38–KRd+ASCT (90.7)<br>Anti-CD38–VRd+ASCT (90.5)	**1.70 (1.22–2.37)**
Excluding GMMG-HD7 (MRD before ASCT)	16 (5,633)	0.23	Anti-CD38–VRd+ASCT (93.8)<br>Anti-CD38–KRd (88.6)<br>Anti-CD38–KRd+ASCT (87.4)	**1.71 (1.08–2.71)**
10^{-5} threshold only (excluding IFM 2009)	16 (5,784)	0.41	Anti-CD38–KRd (87.9)<br>Anti-CD38–VRd+ASCT (87.3)<br>Anti-CD38–KRd+ASCT (85.0)	1.81 (0.68–4.81)
Excluding abstract-only data (ADVANCE, COBRA, AGMT-MM04)†	14 (5,668)	0.26	Anti-CD38–KRd+ASCT (94.3)<br>Anti-CD38–KRd (86.6)<br>KRd+ASCT (81.7)	**1.72 (1.04–2.82)**
TE / ASCT-deferred trials only	10 (3,814)	0.30	Anti-CD38–KRd (90.7)<br>Anti-CD38–VRd+ASCT (83.7)<br>Anti-CD38–KRd+ASCT (78.6)	1.72 (1.00–2.94)
TI trials only†	7 (2,479)	0.11	Anti-CD38–KRd (96.8)<br>KRd (83.4)<br>VMP→Rd (59.2)	–
Separate daratumumab and isatuximab nodes	17 (6,293)	0.21	Dara–VRd+ASCT (96.1)<br>Dara–KRd (87.0)<br>Isa–KRd+ASCT (85.9)	**1.71 (1.10–2.67)**
**Leave-one-trial-out**				
Without PERSEUS	16 (5,584)	0.39	Anti-CD38–KRd (89.5)<br>Anti-CD38–KRd+ASCT (86.3)<br>Anti-CD38–VRd+ASCT (84.7)	1.72 (0.90–3.27)
Without IMROZ	16 (5,847)	0.41	Anti-CD38–KRd (88.2)<br>Anti-CD38–VRd+ASCT (86.9)<br>Anti-CD38–KRd+ASCT (85.2)	1.72 (0.89–3.32)
Without MAIA	16 (5,556)	0.30	Anti-CD38–VRd+ASCT (90.9)<br>Anti-CD38–KRd (88.2)<br>Anti-CD38–KRd+ASCT (86.4)	1.72 (1.00–2.94)

Analysis	Trials (patients)	τ	Three top-ranked strategies (SUCRA, %)	VRd+ASCT vs VRd, OR (95% CI)
Without CEPHEUS	16 (5,898)	0.39	Anti-CD38–KRd (88.5)<br>Anti-CD38–VRd+ASCT (87.7)<br>Anti-CD38–KRd+ASCT (86.2)	1.72 (0.90–3.27)
Without GMMG-HD7	16 (5,633)	0.23	Anti-CD38–VRd+ASCT (94.0)<br>Anti-CD38–KRd (88.5)<br>Anti-CD38–KRd+ASCT (87.4)	**1.71 (1.08–2.71)**
Without GRIFFIN	16 (6,086)	0.34	Anti-CD38–KRd (89.7)<br>Anti-CD38–KRd+ASCT (87.3)<br>Anti-CD38–VRd+ASCT (85.6)	1.72 (0.95–3.09)
Without IsKia	16 (5,991)	0.35	Anti-CD38–KRd (92.8)<br>Anti-CD38–VRd+ASCT (91.8)<br>KRd+ASCT (75.9)	1.72 (0.95–3.10)
Without DETERMINATION	16 (6,095)	0.41	Anti-CD38–KRd (88.5)<br>Anti-CD38–VRd+ASCT (86.0)<br>Anti-CD38–KRd+ASCT (85.5)	1.65 (0.67–4.04)
Without IFM 2009	16 (5,784)	0.41	Anti-CD38–KRd (87.9)<br>Anti-CD38–VRd+ASCT (87.0)<br>Anti-CD38–KRd+ASCT (85.1)	1.81 (0.68–4.81)
Without COBRA†	16 (6,043)	0.33	Anti-CD38–KRd (92.6)<br>Anti-CD38–KRd+ASCT (91.1)<br>KRd+ASCT (78.5)	1.72 (0.98–3.02)
Without BENEFIT	16 (6,023)	0.33	Anti-CD38–VRd+ASCT (90.6)<br>Anti-CD38–KRd (87.7)<br>Anti-CD38–KRd+ASCT (85.3)	1.72 (0.98–3.02)
Without ADVANCE	16 (6,006)	0.30	Anti-CD38–VRd+ASCT (90.8)<br>Anti-CD38–KRd+ASCT (89.0)<br>Anti-CD38–KRd (79.4)	1.72 (1.00–2.95)
Without CARDAMON	16 (6,075)	0.35	Anti-CD38–KRd (88.3)<br>Anti-CD38–VRd+ASCT (87.0)<br>Anti-CD38–KRd+ASCT (85.8)	1.72 (0.95–3.10)
Without EMN20	16 (6,211)	0.33	Anti-CD38–VRd+ASCT (90.5)<br>Anti-CD38–KRd (87.6)<br>Anti-CD38–KRd+ASCT (85.7)	1.72 (0.98–3.02)
Without GEM2017FIT	16 (5,832)	0.30	Anti-CD38–KRd (94.0)<br>Anti-CD38–VRd+ASCT (86.1)<br>Anti-CD38–KRd+ASCT (84.3)	1.72 (1.00–2.95)

Analysis	Trials (patients)	τ	Three top-ranked strategies (SUCRA, %)	VRd+ASCT vs VRd, OR (95% CI)
Without AGMT-MM04	16 (6,205)	0.32	Anti-CD38–KRd (89.6)<br>Anti-CD38–VRd+ASCT (88.5)<br>Anti-CD38–KRd+ASCT (87.1)	1.72 (0.98–2.99)

ASCT, autologous stem-cell transplantation; Dara, daratumumab; Isa, isatuximab; K, carfilzomib; d, dexamethasone; OR, odds ratio; R, lenalidomide; SUCRA, surface under the cumulative ranking curve; TE, transplant-eligible; TI, transplant-ineligible; V, bortezomib; VMP→Rd, bortezomib–melphalan–prednisone followed by lenalidomide–dexamethasone; τ, between-study standard deviation.

Supplementary S6 – Risk-of-bias assessment

	Random sequence generation (selection bias)	Allocation concealment (selection bias)	Blinding of participants and personnel (performance bias)	Blinding of outcome assessment (detection bias)	Incomplete outcome data (attrition bias)	Selective reporting (reporting bias)	Other bias
ADVANCE	+	+	+	+	?	+	?
AGMT-MM04	?	?	+	+	−	?	?
BENEFIT	+	+	+	+	+	+	+
CARDAMON	+	+	+	+	+	+	+
CEPHEUS	+	+	+	+	+	+	+
COBRA	+	+	+	+	?	+	+
DETERMINATION	+	+	+	+	−	+	+
EMN20	+	?	+	+	+	+	+
FORTE	+	+	+	+	+	+	+
GEM2017FIT	+	?	+	+	+	+	+
GMMG-HD7	+	+	+	+	+	+	+
GRIFFIN	+	+	+	+	+	+	+
IFM 2009	+	+	+	+	?	+	+
IMROZ	+	+	+	+	+	+	+
ISKIA	+	+	+	+	+	+	+
MAIA	+	+	+	+	+	+	+
PERSEUS	+	+	+	+	+	+	+

Supplementary S7 – CINeMA

Comparison	Direct trials, n	OR (95% CI)	Within-study bias	Reporting bias	Indirectness	Imprecision	Heterogeneity	Incoherence	Confidence	Reasons for downgrading
**Each strategy vs VRd**										
Anti-CD38–KRd vs VRd	0	4.77 (1.67–13.63)	Some concerns	Low risk	No concerns	No concerns	No concerns	Some concerns	**Low**	Within-study bias, Incoherence
Anti-CD38–VRd+ASCT vs VRd	0	4.87 (2.31–10.28)	Some concerns	Low risk	No concerns	No concerns	No concerns	Some concerns	**Low**	Within-study bias, Incoherence
Anti-CD38–KRd+ASCT vs VRd	0	4.69 (1.09–20.24)	No concerns	Low risk	No concerns	No concerns	Major concerns	Some concerns	**Very low**	Heterogeneity, Incoherence
KRd+ASCT vs VRd	0	2.86 (0.87–9.41)	No concerns	Low risk	No concerns	Some concerns	Some concerns	Some concerns	**Very low**	Imprecision, Heterogeneity, Incoherence
KRd vs VRd	1	2.42 (1.01–5.80)	Some concerns	Low risk	Some concerns	No concerns	Major concerns	No concerns	**Very low**	Within-study bias, Indirectness, Heterogeneity
Anti-CD38–VRd vs VRd	2	1.95 (1.13–3.38)	No concerns	Low risk	No concerns	No concerns	Major concerns	No concerns	**Low**	Heterogeneity
VRd+ASCT vs VRd	2	1.72 (0.95–3.10)	Some concerns	Low risk	Some concerns	Some concerns	Some concerns	Some concerns	**Very low**	Within-study bias, Indirectness, Imprecision, Heterogeneity, Incoherence
KCd+ASCT vs VRd	0	1.49 (0.45–4.92)	No concerns	Low risk	No concerns	Major concerns	No concerns	Some concerns	**Very low**	Imprecision, Incoherence
VMP→Rd vs VRd	0	0.90 (0.28–2.91)	Some concerns	Low risk	No concerns	Major concerns	No concerns	Some concerns	**Very low**	Within-study bias, Imprecision, Incoherence
KCd vs VRd	0	0.71 (0.16–3.12)	No concerns	Low risk	No concerns	Major concerns	No concerns	Some concerns	**Very low**	Imprecision, Incoherence

Anti-CD38–Rd vs VRd	0	0.54 (0.20–1.43)	No concerns	Low risk	No concerns	Major concerns	No concerns	Some concerns	**Very low**	Imprecision, Incoherence
Rd vs VRd	0	0.10 (0.03–0.36)	No concerns	Low risk	No concerns	No concerns	No concerns	Some concerns	**Moderate**	Incoherence
**With vs without ASCT**										
VRd+ASCT vs VRd	2	1.72 (0.95–3.10)	Some concerns	Low risk	Some concerns	Some concerns	Some concerns	Some concerns	**Very low**	Within-study bias, Indirectness, Imprecision, Heterogeneity, Incoherence
KRd+ASCT vs KRd	1	1.18 (0.52–2.66)	No concerns	Low risk	No concerns	Major concerns	No concerns	Some concerns	**Very low**	Imprecision, Incoherence
KCd+ASCT vs KCd	1	2.10 (0.87–5.05)	No concerns	Low risk	No concerns	Some concerns	Some concerns	Some concerns	**Very low**	Imprecision, Heterogeneity, Incoherence
Anti-CD38–VRd+ASCT vs Anti-CD38–VRd	0	2.50 (0.99–6.32)	No concerns	Low risk	No concerns	Some concerns	Some concerns	Some concerns	**Very low**	Imprecision, Heterogeneity, Incoherence
Anti-CD38–KRd+ASCT vs Anti-CD38–KRd	0	0.98 (0.27–3.64)	No concerns	Low risk	No concerns	Major concerns	No concerns	Some concerns	**Very low**	Imprecision, Incoherence
**With vs without anti-CD38 (same backbone)**										
Anti-CD38–VRd+ASCT vs VRd+ASCT	3	2.84 (1.80–4.48)	No concerns	Low risk	No concerns	No concerns	No concerns	Some concerns	**Moderate**	Incoherence
Anti-CD38–KRd+ASCT vs KRd+ASCT	1	1.64 (0.70–3.83)	No concerns	Low risk	No concerns	Major concerns	No concerns	Some concerns	**Very low**	Imprecision, Incoherence
Anti-CD38–VRd vs VRd	2	1.95 (1.13–3.38)	No concerns	Low risk	No concerns	No concerns	Major concerns	No concerns	**Low**	Heterogeneity
Anti-CD38–KRd vs KRd	2	1.97 (1.10–3.52)	Some concerns	Low risk	No concerns	No concerns	Major concerns	Some concerns	**Very low**	Within-study bias, Heterogeneity,

										Incoherence
Anti-CD38–Rd vs Rd	2	5.21 (2.37–11.44)	No concerns	Low risk	No concerns	No concerns	No concerns	No concerns	**High**	
**Among the three top-ranked strategies**										
Anti-CD38–KRd vs Anti-CD38–VRd+ASCT	0	0.98 (0.27–3.53)	Some concerns	Low risk	No concerns	Major concerns	No concerns	Some concerns	**Very low**	Within-study bias, Imprecision, Incoherence
Anti-CD38–KRd vs Anti-CD38–KRd+ASCT	0	1.02 (0.27–3.76)	No concerns	Low risk	No concerns	Major concerns	No concerns	Some concerns	**Very low**	Imprecision, Incoherence
Anti-CD38–VRd+ASCT vs Anti-CD38–KRd+ASCT	0	1.04 (0.20–5.35)	No concerns	Low risk	No concerns	Major concerns	No concerns	Some concerns	**Very low**	Imprecision, Incoherence
